# Ecological connectivity networks in rapidly expanding cities

**DOI:** 10.1016/j.heliyon.2017.e00325

**Published:** 2017-06-23

**Authors:** Amal Najihah M. Nor, Ron Corstanje, Jim A. Harris, Darren R. Grafius, Gavin M. Siriwardena

**Affiliations:** a School of Water, Energy and Environment, Cranfield University, MK43 0AL, Bedford, UK; b Faculty of Earth Science, University Malaysia Kelantan, Jeli Campus, 17600, Kelantan, Malaysia; c Land-Use Research, British Trust for Ornithology, The Nunnery, Thetford, Norfolk IP24 2PU, UK

**Keywords:** Ecology

## Abstract

Urban expansion increases fragmentation of the landscape. In effect, fragmentation decreases connectivity, causes green space loss and impacts upon the ecology and function of green space. Restoration of the functionality of green space often requires restoring the ecological connectivity of this green space within the city matrix. However, identifying ecological corridors that integrate different structural and functional connectivity of green space remains vague. Assessing connectivity for developing an ecological network by using efficient models is essential to improve these networks under rapid urban expansion. This paper presents a novel methodological approach to assess and model connectivity for the Eurasian tree sparrow (*Passer montanus*) and Yellow-vented bulbul (*Pycnonotus goiavier*) in three cities (Kuala Lumpur, Malaysia; Jakarta, Indonesia and Metro Manila, Philippines). The approach identifies potential priority corridors for ecological connectivity networks. The study combined circuit models, connectivity analysis and least-cost models to identify potential corridors by integrating structure and function of green space patches to provide reliable ecological connectivity network models in the cities. Relevant parameters such as landscape resistance and green space structure (vegetation density, patch size and patch distance) were derived from an expert and literature-based approach based on the preference of bird behaviour. The integrated models allowed the assessment of connectivity for both species using different measures of green space structure revealing the potential corridors and least-cost pathways for both bird species at the patch sites. The implementation of improvements to the identified corridors could increase the connectivity of green space. This study provides examples of how combining models can contribute to the improvement of ecological networks in rapidly expanding cities and demonstrates the usefulness of such models for biodiversity conservation and urban planning.

## 1 Introduction

In urban systems, green spaces play a key role in conserving biodiversity in a sustainable landscape by providing habitat, food sources and connectivity between groups which otherwise would be isolated by the urban matrix [1, 2]. However, green space is increasingly encroached upon and fragmented as cities’ population density increases [3]. The proportion of the world’s population living in cities is expected to surpass 65% by 2025 [4], and population increases are accompanied by intensified urban development. As a result, urban expansion has increased fragmentation in the landscape and has eliminated green space, particularly dispersal corridors [5]. In this regard, fragmentation decreases connectivity, increasing isolation of habitats and green space loss [5]. Therefore, conservation of green space connectivity through ecological networks in rapidly expanding cities is needed to protect the biodiversity in urban area.

The term ‘ecological network’ is defined as a network composed of ecological components such as core areas, ecological corridors and buffer zones [6]. These components contain natural, semi-natural or restored vegetation and are configured and managed to allow the sustainable use of natural resources and to conserve biodiversity [7]. Such networks play the role of corridors for wildlife species to sustain healthy populations. Ecological networks can provide a solution to the problems of intensified land use and fragmentation, enabling natural populations of species and threatened habitats to survive [3]. Despite the increased research in landscape connectivity in conservation planning in rural areas, there is a limited number of such studies in urban areas [2, 8, 9]. Therefore, the development of ecological networks is increasingly considered a suitable approach to improve the ecological function of green space in the urban landscape [2, 5].

In landscape ecology, connectivity (corridors) is used to describe a landscape’s structural and functional continuity in space and time [10]. Landscape-level habitat connectivity plays an important role in population viability by maintaining the gene flow and facilitating movement, migration, dispersal, distribution and recolonisation [11]. In particular, the landscape-scale spatial configuration and distribution of habitats determine species survival and persistence [12]. Establishing and maintaining connectivity among patches is essential in conserving biodiversity [5]. Furthermore, while urban greening is a key element in sustainable urban development, biodiversity must be an integral component of this greening [5]. Consequently, preserving habitat and dispersal routes and developing a comprehensive ecological network that can maintain landscape-scale connectivity have become crucial factors in urban biodiversity conservation [13, 14]. The development of ecological networks includes protection of existing green spaces, the creation of new spatial forms, restoration and maintenance of connectivity among green space patches [5]. However, only few current analytical tools comprehensively identify potential corridors in regional landscapes under rapid urban expansion [9, 15]. Connectivity models that offer an understanding of the different patterns of functional connectivity under rapid urban expansion are less studied [16]. Planners generally consider only distances between habitat patches [5], not the spatial, ecological or other landscape factors to model the integrated structural and functional connectivity of the landscape [17]. To maintain or restore connectivity, planners must identify the best habitat and potential corridors by quantifying the landscape characteristics such as distances, size and density and consider landscape resistance and the barriers between habitats posed by the landscape and land use [18].

Current models use Euclidean distance, connectivity indices, least-cost path, least-cost distance and landscape resistance using circuit theory to model connectivity [19, 20, 21] in a very complex urban landscape. This study considers landscape resistance and green space structure linked to the behaviour of species as parameters and indicators for movement along corridors. Developing landscape connectivity models using circuit theory parameterised with green space structure characteristics such as size and density allows the modelling of multiple paths between nodes [22]. The use of circuit theory to depict spatial patterns of landscape resistance or conductance provides an easily interpretable method for calculating metric values and modelled linkages [23]. Apart from that, least-cost path analysis represents a valuable method for conservation planning by analysing and designing habitat corridors [24]. It allows quantitative comparisons of potential movement routes over large study areas, can incorporate simple or complex models of habitat effects on movement and influences functional connectivity for species movement [25]. In this study, we propose the identification of potential corridors in the cities using circuit theory, connectivity analysis and least-cost path models to develop potential corridors and can improve ecological networks, so planners can identify the relative high-quality habitats and choose the best opportunities to maintain and restore connectivity. The ecological network developed based on these integrated models simplified and systematised the complex landscape, helping to identify the significance of each green space and guiding urban planning for biodiversity conservation [5].

This study presents a novel integrated approach to assess and model connectivity for two bird species Eurasian tree sparrow (*Passer montanus*) and Yellow-vented bulbul (*Pycnonotus goiavier*). The Eurasian tree sparrow is found most abundantly and is the dominant species in the urban areas and one of the most common birds found in a variety of environments in Southeast Asia [26, 27, 28]. Yellow-vented bulbul (*Pycnonotus goiavier*) is a song bird species and the second most abundant in Southeast Asia and are found predominantly in lowland disturbed habitats such as scrub forest edge, other plantations and garden habitats [26, 28]. We chose a multi-species approach representing different groups of birds that are partially adapted to urban areas (Yellow-vented bulbul) and well adapted in urban areas (Eurasian tree sparrow) as a surrogate model for identifying priority corridors [14]. Eurasian tree sparrow (*Passer montanus*) is an urban commensal in tropical Asia and can survive in highly built-up areas, while Yellow-vented bulbul (*Pycnonotus goiavier*) presence is more correlated with green cover in the landscape [29]. Both species are urban tolerant but require green cover for breeding success. Urban green spaces appear to suit them best by providing nesting trees and food resources [29]. The abundance and distribution of these species is likely to be affected by habitat and food losses in highly urbanised areas [27, 30, 31]. For these reasons, the aim of this study is to develop an ecological landscape connectivity network in three cities under rapid expansion in Southeast Asia to conserve critical green space patches and to provide an initial guideline for urban planning.

## 2 Materials and methods

### 2.1 Study area

**Fig. 1:**
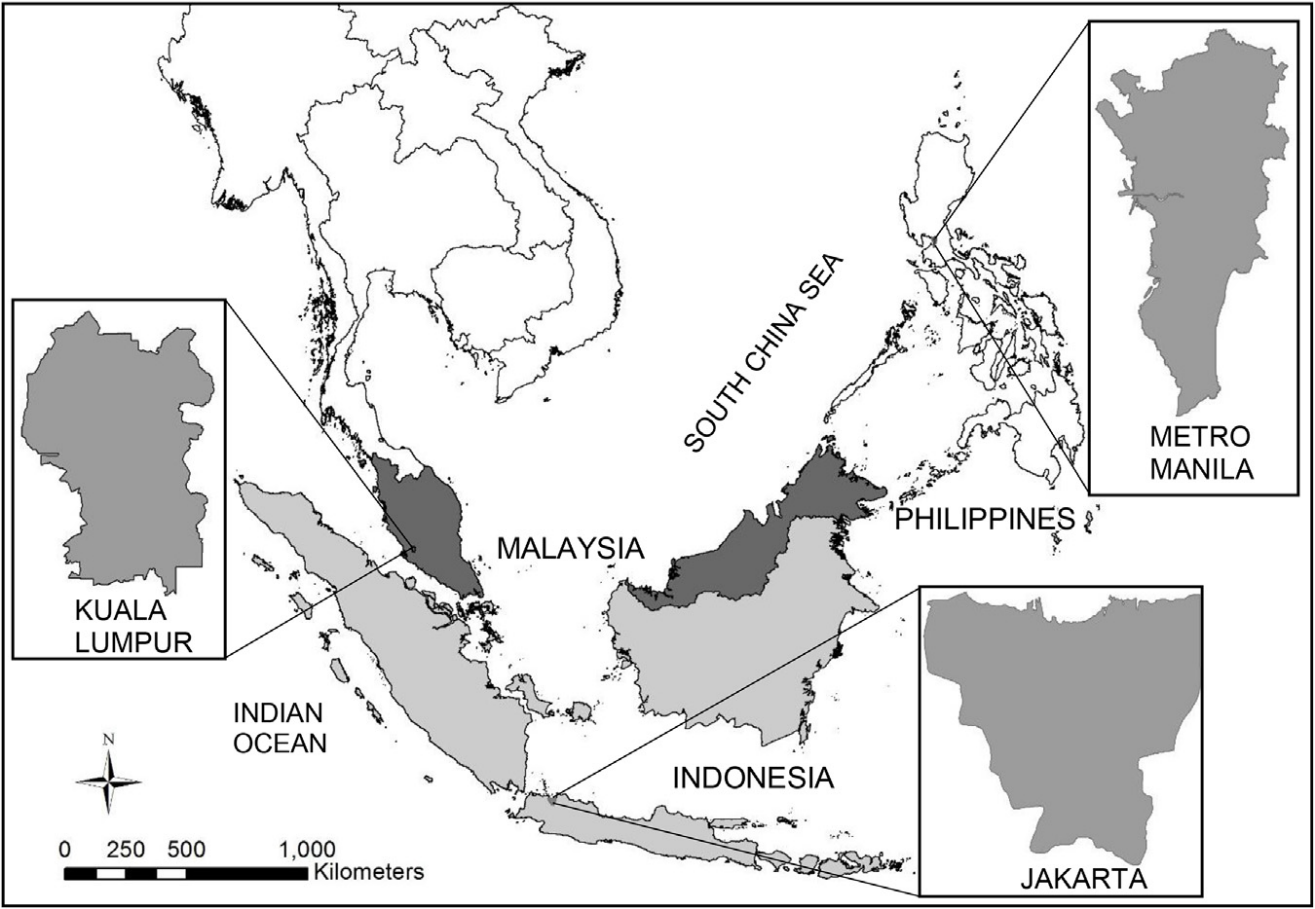
Location map of three cities in Southeast Asia.

This study focussed on three Southeast Asian cities; Kuala Lumpur, Malaysia; Jakarta, Indonesia and Metro Manila, Philippines (Fig. 1). These cities were chosen due to their rapid expansion and the emergence of urban regions [32]. The high pace of rapid urban development, population growth and economic growth in these cities has accelerated the increase of environmental and habitat degradation [32].

Kuala Lumpur, the capital of Malaysia, is located at the confluence of the Klang and Gombak rivers and its total area is approximately 23934 ha (239 km^2^). Jakarta, the capital of Indonesia, consists of five municipalities and lies in the lowland on the northwest coast of Java Island. The city occupies an area of 64000 ha (640 km^2^). Jakarta has a flat terrain, and the land gradually rises from 5 to 50 m above mean sea level [33]. Metro Manila, the capital of the Philippines consists of eight contiguous cities, including Manila city, and nine other municipalities, covering an area of approximately 63800 ha (638 km^2^). The capital is located in the lowlands of Southwestern Luzon Island on the eastern coast of Manila Bay [33].

There are plants that provide food resource and shelter for various bird species in Southeast Asia [34]. For example, *Artocarpus bilimbi* and *Sandirum koetjape* are food plants for frugivores and granivores birds that eat fruits and seed while *Gardenia carinata* as a food plant for nectar eaters. *Spathodea campanulata* for nectarivores bird species and *Psidum guajava* for omnivores bird species that attract insects. There are several trees for shelter and shrub nester are *Adenanthera pavonina*, *Canaga odorata*, *Cinnanomum iners*, *Cerbera odollam* and *Alstonia angustiloba* [34].

However, in Kuala Lumpur, there are more exotic and ornamental plants such as palm trees that have been planted compared to food plants [34]. In Jakarta, *Pterecarpus indica* is the predominant roadside tree species, some flowering shrubs and palm trees in the medians of roads [35]. Nine tree species were found to be the most common in Jakarta, *Canarium indicum, Tamarindus indica, Khaya senegalensis, Ficus lyrata, Artocarpus integer, Samanea saman, Areca catechu, Mangifera indica, Tamarindus indica* and *Cocos nucifera* [35]. In Metro Manila, fruit tree, ornamental tree or shade tree such as fruit tree banana (*Moringa oleifera*) is the dominance fruit plant species in the city [26].

### 2.2 Connectivity modelling

**Fig. 2:**
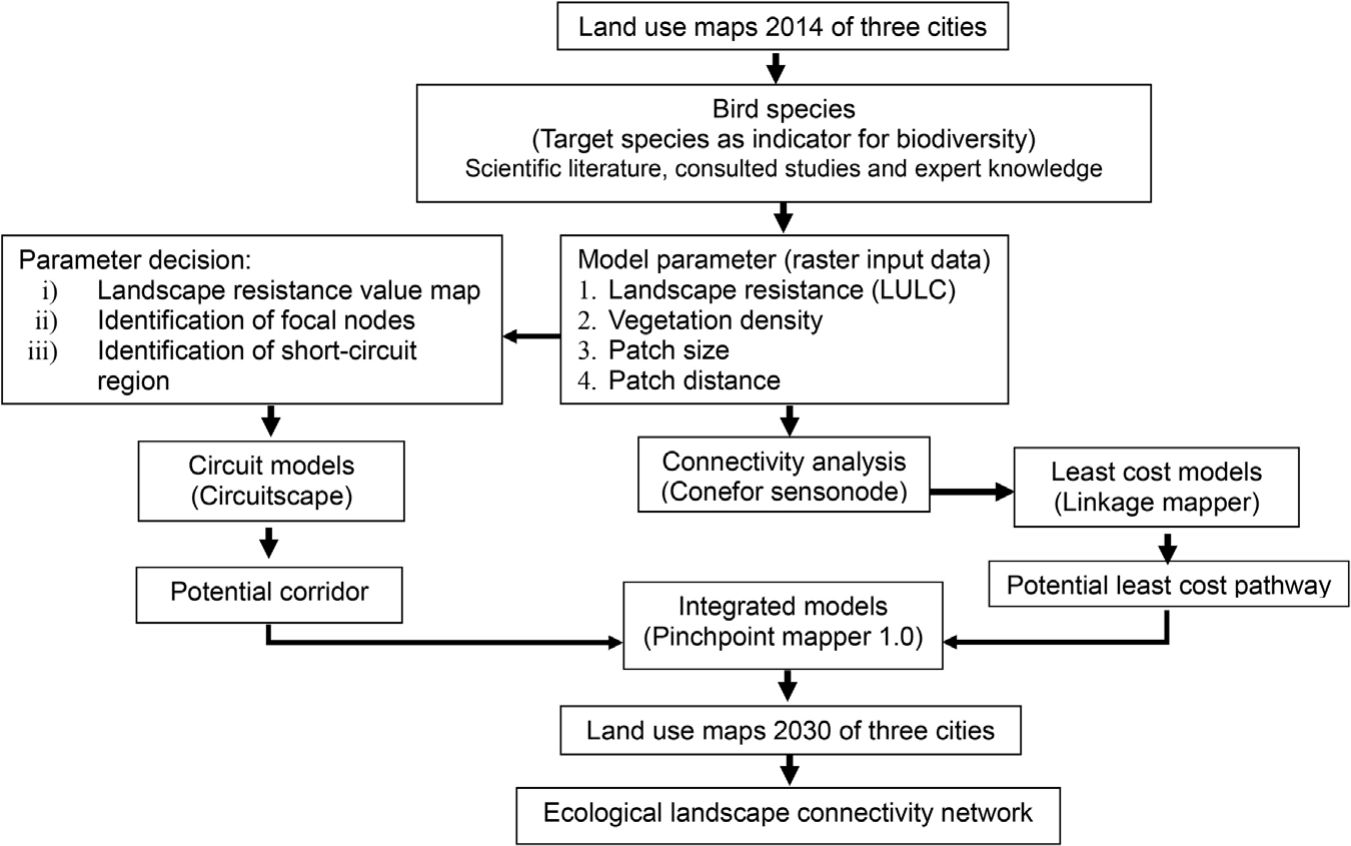
Methodological framework.

The methodological framework for modelling potential corridors involved: (1) modelling a resistance surface for Eurasian tree sparrow (*Passer montanus*) and Yellow-vented bulbul (*Pycnonotus goiavier*) based on the selected parameters; (2) modelling hypothetical dispersal corridors from the resistance surface models using circuit theory analyses with patch sites for both species; and (3) identifying the priority corridors and assessing their connectivity by combining circuit models, connectivity analysis and least-cost modelling (Fig. 2). In this study, circuits are defined as networks of nodes connected by resistors (electrical components that conduct current, voltage and resistance) [22] and were used to represent potential corridor maps. Connectivity analysis was conducted to calculate linear distance metrics between nodes to be used in least-cost path models. The least-cost paths were calculated to represent the route of maximum efficiency between two locations [24] as a function of the distance travelled and the costs traversed. These analyses were chosen for their simple, easy-to-apply approach, computable and capable of handling various data while avoiding excessive and unnecessary complexity.

### 2.3 Target species

The level of connectivity in a landscape varies among environments, but most of all among species [13]. Depending on the species, a landscape will be perceived differently [36] and may provide different levels of connectivity [13]. We chose birds as target species because they are visible, less secretive and easy to find within the open space [37]. Birds are indicators of abundance of biodiversity inhabiting an urban area and provide an indicator for ecological functions of green space such as seed dispersal [9]. Bird dispersal is considered a major driver of plant community dynamics over time and important in increasing the vegetation cover in the urban landscape [38, 39]. Specifically, frugivorous and granivorous birds constitute the most effective mobile links for connecting habitats, acting as dispersers of seeds from fruit plants, off-plant and the ground [39, 40]. These mobile organisms can provide connectivity between isolated areas in fragmented environments [38]. Consequently, several studies on urban landscapes have been carried out using bird species as indicators of habitat quality [17, 41, 42]. The richness and abundance of bird species are important for ecological and well-being of urban environment and therefore conservation of priority habitat and the functional networks of urban green space for these species are needed to support ecological sustainability and conservation of biodiversity [12].

The selected species represent different functional guilds and habitat preference that help to link the structure and functional connectivity of green space [37]. Both species are important seed dispersers in urban areas which makes them suitable model species to study the conservation priorities closely linked to ecological and human environments [38]. We chose the Eurasian tree sparrow (*Passer montanus*) because of its functional guilds (granivorous) that eat small grains or seeds and disperse them from the ground or off plants [27]. This species disperse seeds at the 1 km scale [43]. They are ground nesters, secondary cavity nesters and exotic species [27, 44]. For nesting sites, the habitat occupancy of the Eurasian tree sparrow (*Passer montanus*) is frequent in urban areas where it chooses trees and they nest in hollows of buildings [31].

Yellow-vented bulbul (*Pycnonotus goiavier*) is also an adapted urban bird but it prefers habitat covered by vegetation [45]. They are shrub nesters and omnivores [27] that search for food predominantly on the ground or in low-lying vegetation [46]. They are frugivorous (fruit eaters) which typically take fruits from a perch, swallow them whole and seeds are defecated or regurgitated at open sites [47]. This species is the second most important as a feeder on the fruits in the tree canopy and dispersing seeds 50 to 100 m from their parent tree and moving in the range of 1 to 1250 m [48]. They are also insectivore bird species that help plants by controlling the quantity of insects living on bark, leaves and branches [34]. They forage within tree foliage and adjust their breeding activities and foraging areas by tracking food resources within 500 m of their nesting sites [28].

Although neither of these bird species are currently considered to be threatened or otherwise a conservation priority, they both act as important indicators and drivers of urban ecosystem health as described above. It is for this reason we have selected these species for analysis.

### 2.4 Model parameters

Four parameters representing the behaviour of the two focal species were identified: i) landscape resistance (based on land use land cover (LULC) types), ii) vegetation density (foraging and nesting), iii) patch size (nesting sites and breeding), and iv) patch distance (seed dispersal). These parameters were derived from an expert opinion and literature-based approach [49].

### 2.5 Landscape resistance values

**Fig. 3:**
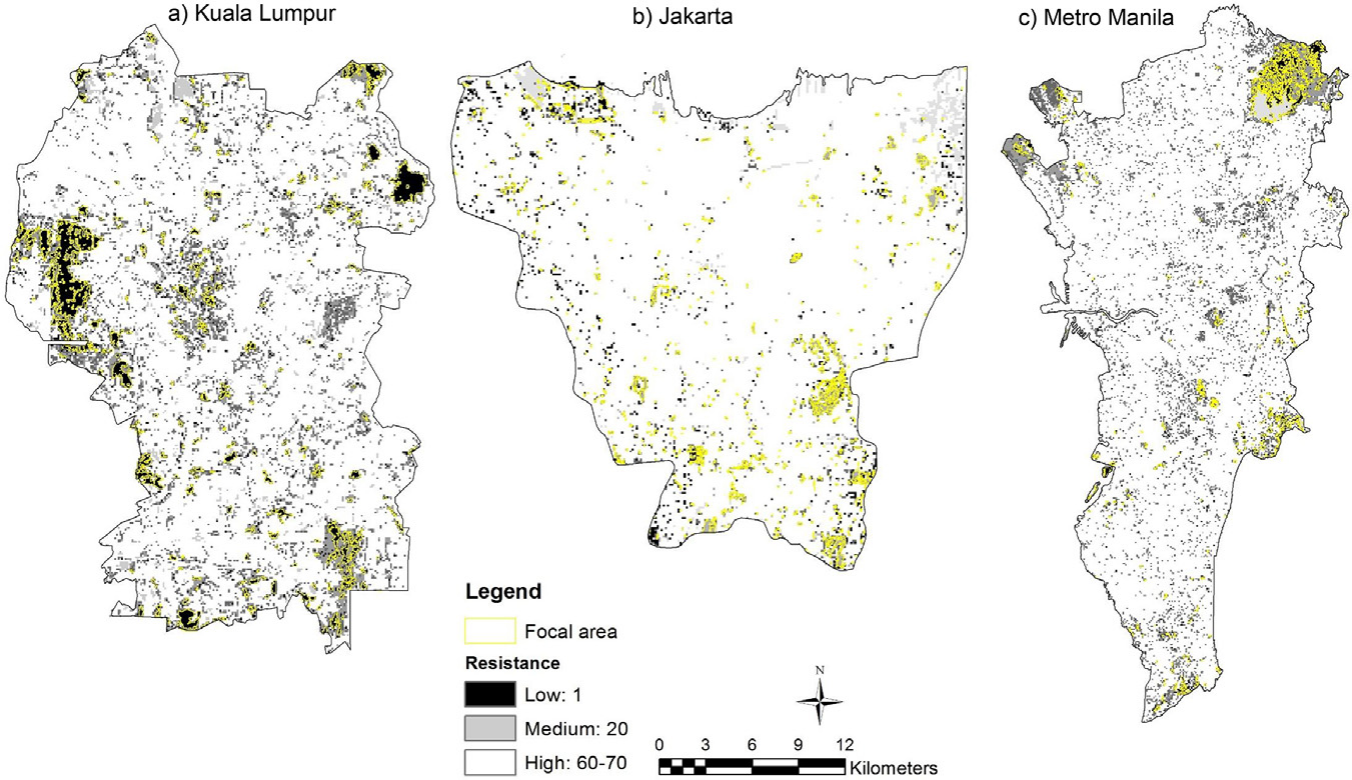
Landscape resistances for Eurasian tree sparrow (*Passer montanus*) within the focal area. Resistance values range from 1 (black) to 100 (white) in a) Kuala Lumpur, b) Jakarta and c) Metro Manila.

**Fig. 4:**
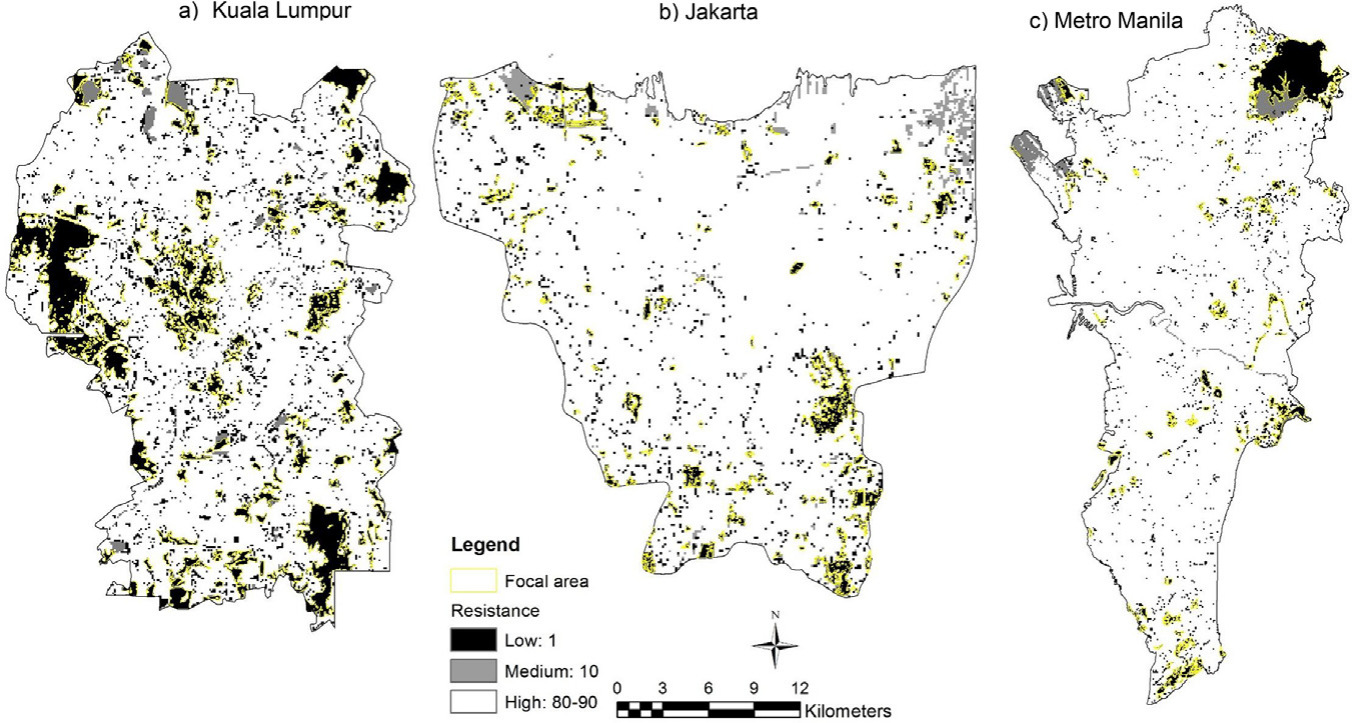
Landscape resistances for Yellow-vented bulbul (*Pycnonotus goiavier*) within the focal area. Resistance values range from 1 (black) to 100 (white) in a) Kuala Lumpur, b) Jakarta and c) Metro Manila.

The resistance surface models were used in circuit analysis to generate maps of movement resistance for both bird species using LULC maps 2014 of three cities in Southeast Asia (derived from [50]). The resistance values ranged from 1 to 100 with the highest resistances mainly related to the presence of built up area. However, both species have different habitat preferences (Tables 1 and 2 ), hence, highly suitable areas are located on the borders of the resistance surface, due to the presence of nesting sites, green space and breeding sites surrounding the cities (Figs. 3 and 4 ).

**Table 1:** Landscape resistance value for Eurasian tree sparrow (*Passer montanus*).

LULC	Resistance value	Justification
Green space	1	Usually forages on the ground and on trees [15].
Built up area	60	Most abundant in development areas, less found in new growth areas and not found in the forest reserves [30]. The abundance of human-associated species increase as the amount of building cover increased [15]. Feeding guild (granivores) was higher in developed areas and sometimes found in areas with greater intensity of land use [30].
Road	70	Along with all routes, most birds were observed in trees and appeared to be either foraging, nesting or singing, with little evidence of the routes being used as flyways [42].
Waterbody	20	Marked preference for breeding sites adjacent to aquatic habitats over sites on farmland associated with wetland habitats, breeding season preference for areas containing water bodies [43].

**Table 2:** Landscape resistance value for Yellow-vented bulbul (*Pycnonotus goiavier*).

LULC	Resistance value	Justification
Green space	1	Species abundance increased when vegetation cover increased. Nest in urban gardens; arboreal and make untidy, cup-shaped nests in trees [28, 45].
Built up area	90	Rarely found in non-vegetation areas [28].
Road	80	Recognises only dense trees, lower tree fractions equal to no trees [42]. Prefer denser trees but can traverse non-tree as last resort [42].
Waterbody	10	Yellow-vented bulbul (*Pycnonotus goiavier*) was recorded the highest densities in the open waterbody habitat [51].

### 2.6 Identification of focal nodes

Focal nodes represent the key habitat patches of interest on the landscape between which flows were modelled in circuit analysis [22]. In the interest of computational feasibility, it is advised to not treat every occurrence of suitable habitat on the landscape as a focal node [52]. For example, green space structure is the important aspect of habitat heterogeneity; it affects bird community structure and enhances bird species diversity [36, 53, 54]. There are different focal areas used for both species. For the Eurasian tree sparrow (*Passer montanus*) the focal nodes are based on patch size, while for Yellow-vented bulbul (*Pycnonotus goiavier*), the focal node used is vegetation density. In this study, patch size refers to green space patch size in unit ha [30], while the vegetation density describes the greenness of the vegetation based on the vegetation index NDVI [55]. The reasoning of focal node selection is presented below and also in Tables 3 and 4 . The parameters for focal areas used in this study include:

**Table 3:** Weight for each parameter and related input layers for the Eurasian tree sparrow (*Passer montanus*).

Parameter (Green space structure)	Weight	Eurasian tree sparrow (*Passer montanus*)			
		Bird nesting site	Seed dispersal	Diet/Feeding/Foraging	Breeding
Habitat patch size	1: < 1 ha 2: 1 to 2 ha 3: 2 to 3 ha 4: 3 to 4 ha 5: 4 to 5 ha 6: > 5 ha	Most species successfully colonised large patches more than smaller ones [30]. Population density decreased with smaller habitat patch area [30].	Larger parks tend to support more diverse habitats and tree species, and have reduced edge effects, which help birds to establish larger, and thus more stable populations [53].	Non-random preferences for foraging habitats [43]. Lower found in larger areas of lawns under the canopy because of more intensive human management and disturbance [43].	The area covered with bush layer, tree layer and pond, >0.05 ha [30]. Larger parks with more visitors could support more omnivores in the breeding season [30]. Increasing random extinction with decreasing habitat size [57].
Patch distance	Maximum distance 1000 m	Seed food within 1 km of the nest-site influenced nest-site choice or affected productivity [43].	All tree fractions equally suitable; avoids gaps [42].	Birds choose the least-cost (optimum) path, encounter fewer hazards, would spend less time in traveling, and travel through habitat with higher probability of containing food and cover [42].	The importance of seed and food resources to the persistence of Eurasian tree sparrow populations. Operates on a larger spatial scale due to the greater mobility in the non-breeding season [43].

**Table 4:** Weight for each parameter and related input layers for Yellow-vented bulbul (Song bird) *Pycnonotus goiavier.*

Parameter (Green space structure)	Weight	Yellow-vented bulbul (Song bird) *Pycnonotus goiavier*			
		Bird nesting site	Seed dispersal	Diet/Feeding/Foraging	Breeding
Vegetation density	1: High density (Trees) 2: M edium density (Shrub) 3: Less density (Grassland)	Nest in urban gardens; arboreal and make untidy, cup-shaped nests in trees. Hole nester (versatile). Strong preference for nest-sites adjacent to wetland habitats, woody vegetation and farmland sites [58].	Fruit 8 to 10 mm, seed deposition, seeds defecated or regurgitated at open sites is limited by perch availability in terms of height, diameter and branching [47].	Forage within tree foliage, which typically take fruits and berries from a perch and swallow them whole, defecating viable seed. High abundance in high vegetation density (woodland) [43].	Adjusting their breeding activities and/or foraging areas by tracking food resources [58].
Patch distance	Maximum distance 1000 m	Nesting site on the trees [58].	All tree fractions equally suitable; avoids gaps within 500-m intervals [42] at distances 10, 20, and 40 m from the border with urban forest to the fringe and 10, 20, 40 and 65 m from urban forest [47].	Birds are assumed to choose the least-cost (optimum) path, encounter fewer hazards, would spend less time in traveling, and travel through habitat with a higher probability of containing food and cover [42].	Small highly isolated patches of forest adversely affect some bird. Nearest distance to waterbody, grassland and trees [58].

#### 2.6.1 a) Patch size

Here, focal nodes were arbitrarily selected as all green space patches greater than 5 ha in size, producing more than 200 of focal nodes for the study areas (Table 3). Green patch size has been demonstrated to be important for urban birds [30], for instance, in the breeding season, the number of species of Eurasian tree sparrow (*Passer montanus*) was affected mainly by park size, with the highest relative importance of 1.00 [30]. Larger parks are easier to move within and have more diverse tree species which could provide various foods [30]. Green patch size was not chosen for Yellow-vented bulbul (*Pycnonotus goiavier*) as Weir and Corlett [48] suggest that patch size has little impact on seed dispersal by these birds and they disperse seeds between a wide range of green patch sizes.

#### 2.6.2 b) Vegetation density

Most of the Yellow-vented bulbul (*Pycnonotus goiavier*) are found in the green space area particularly in high vegetation density [45]. For example, Yellow-vented bulbuls (*Pycnonotus goiavier*) were mostly seen in short or medium height trees [45]. Therefore, vegetation density was chosen for the focal node selection in the circuit analysis. Normalized Difference Vegetation Index (NDVI) derived from remotely sensed images has been used in various studies to distinguish between vegetated and non-vegetated areas [55], thus, NVDI analysis was chosen to detect density in the vegetation cover (Table 4). The NDVI is a graphical indicator that indicates the amount of green biomass in the area [56]. The NDVI was calculated in the ERDAS Imagine software 10.1. Highly vegetated areas have a NDVI value closer to 1, while locations dominated by water, cleared land or bare soil and built up area have values closer to −1. Based on the percentage, each cell was classified based on three vegetation density classes which are high, medium and low vegetation density (see Table 4) on the scale of 0.25 to 1, where less than 50% of the green in a cell was categorised as low vegetation density, and given a value of 0.25. In the same manner, 0.5 (medium vegetation density) and 0.75 to 1 (high vegetation density) values were given to cells where the percentage of green is 25 to 50%, 50 to 75% and more than 75%, respectively. Sandström et al. [37] found that species richness of hole-nesters partially adapted to urban environment (e.g Yellow-vented bulbul) was positively correlated with vegetation density while well-adapted urban birds (e.g Eurasian tree sparrow) showed an inverse correlation. Therefore, the parameter of vegetation density was not used in focal node selection for Eurasian tree sparrow (*Passer montanus*) because it showed no preference for vegetation density [37]. They usually forage on the ground and on trees and have adapted to foraging in garbage. Their breeding site can also be in trees and in low urbanisation areas (low density of building, residential and road areas) [15].

### 2.7 Patch distance for connectivity analysis

A parameter of patch distance was used in the connectivity analysis. Distance factor relates to the behaviour of seed dispersal. Frugivorous (Yellow-vented bulbul) and granivorous (Eurasian tree sparrow) birds are the most important seed dispersal agents for urban tropical forest into grasslands and early successional vegetation because the simple structure of these habitats poses less of a barrier to them [39, 40]. Both species have a dispersal capability of about 1 km [43, 48]. The input of bird-dispersed seeds increases with distance from green space edge more than bat-dispersed seed, presumably because birds are more likely to perch to defecate rather than doing so in flight [47]. Both seed density and number of species were significantly affected by distance from vegetation area (0 to 40 m) [47] (Tables 3 and 4).

### 2.8 Identification of short-circuit regions

Short-circuit regions were used in circuit analysis to represent areas that the organisms under study can traverse freely with no cost [52]. It must be determined if this should be represented by all favourable habitat on the landscape (e.g. all green space cells), only the same areas as the focal nodes (thus treating smaller green space patches as having low resistance but not quite as favourable as focal habitat patches), or if some other criteria would be most appropriate [52]. Here, the same file was used for short-circuit regions as for focal nodes. This was based on the study by Zhou and Chu [30] found that large green patch size is suitable habitats would act as sources and destinations for Eurasian tree sparrow (*Passer montanus*) movement, but smaller patches may act as low-cost corridors for movement between larger habitats rather than sources and destinations in their own right. For Yellow-vented bulbul (*Pycnonotus goiavier*), habitats with high vegetation density provide more food sources, movement and breeding compared to low vegetation density [15].

### 2.9 Circuit models

Circuit models were created using the Circuitscape software [52]. Circuitscape enables consideration of least-cost flow pathways and variable maps of ‘resistance’. Circuitscape is used in the field of landscape ecology to model an organism’s tendency or reluctance to move through certain land cover types that can be mapped in a Geographical Information Systems (GIS). Landscape resistance and patch sites had to be converted into ASCII rasters via the ‘Export to Circuitscape’ extension for ArcGIS 10.2 (ESRI, Redlands, CA) for use in the software. Circuit models for both species were generated using the pairwise mode in order to model the connectivity between all pairs of patch sites [57, 58]. The pairwise operation runs by iteratively testing the ‘current flow’ (i.e. connectivity) between all identified pairs of ‘focal nodes’ (i.e. key habitat patches) in the landscape. When it is run in this way, Circuitscape requires three input datasets: i) input resistance data ii) focal node location files and iii) short-circuit region file. Parameter decisions were made based on: i) landscape resistance values: ii) identification of focal nodes and iii) identification of short-circuit regions. A cumulative current density map was produced that combined the results of all pairwise current density maps.

### 2.10 Connectivity analysis

We calculated patch distance using the Conefor Inputs extension runs in ArcGIS. Sensonode Software [59] embedded in ArcGIS 10.2 was used to generate link ID for both species. The maximum distance value for both species was set to 1000 m distance according to the behavioural factor of maximum dispersal distance (Tables 3 and 4 ; [43]). The extension generates the node and connection files required by Conefor from a vector layer in ArcGIS. Before using this extension, the vector layer must have two fields containing the IDs of the nodes or patches (spatial features, typically polygons) and the attributes of the nodes (e.g. habitat area or any other attribute of interest could be used). The extension generates the node and connection files, with the connections characterised by the Euclidean (straight-line) distance between patches. These distances are calculated either from the edges of the patches (the most typical and generally recommended option) or from the centroids of the patches.

### 2.11 Least-cost models

The tool Linkage Mapper 1.0 [24] generated least-cost models for both species. Landscape resistance was used as cost surfaces together with the patch site polygons and a file comprising calculated distances between patch sites.

### 2.12 Integrated models

Pinchpoint Mapper 1.0 [60], which is part of the Linkage Mapper toolkit, was used to create models combining least-cost and circuit methods. By constraining the current flow to the least-cost corridors identified, the combined method was able to highlight least-cost corridors and to assess the connectivity via the least-cost distance and least-cost path length metrics. Then, by running the Circuitscape software within the least-cost corridors, the tool assessed the connectivity via the effective resistance metric and mapped existing pinchpoints (critical connections) within least-cost corridors.

### 2.13 Ecological connectivity model 2030

To provide an idea of how the connectivity models could be used to improve connectivity for future planning, the combined model was overlaid to the predicted land use map for 2030 (derived from Nor et al., in review) for the ecological connectivity model in 2030.

## 3 Results

### 3.1 Circuit models

**Fig. 5:**
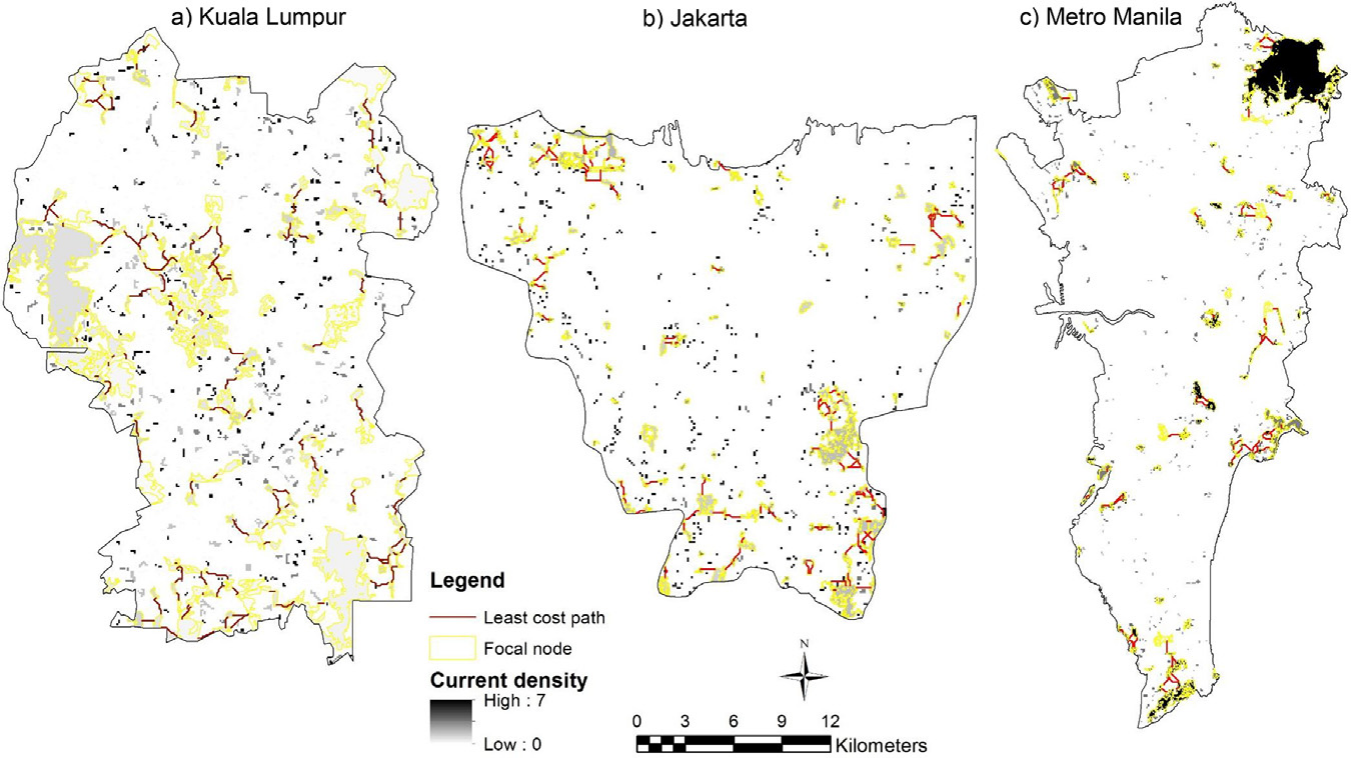
Current density for Eurasian tree sparrow (*Passer montanus*) within focal area in a) Kuala Lumpur, b) Jakarta and c) Metro Manila.

**Fig. 6:**
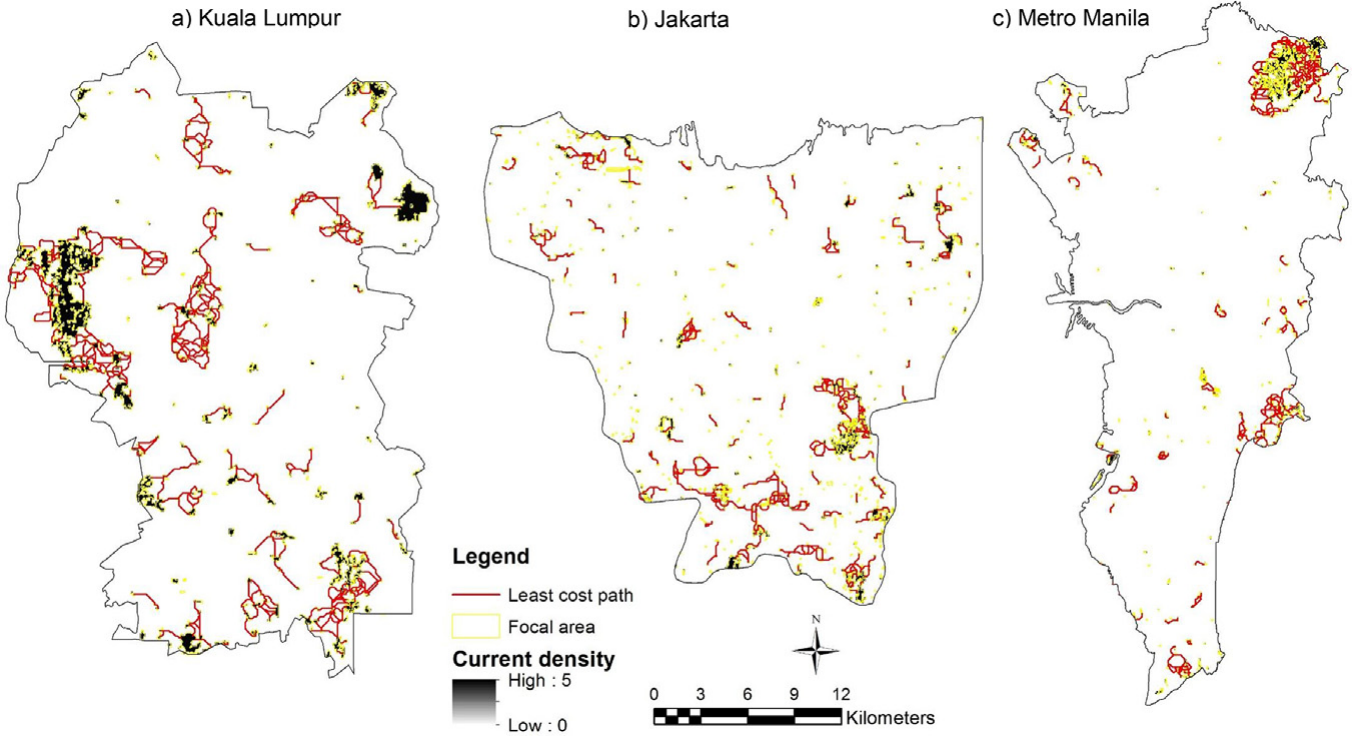
Current density for Yellow-vented bulbul (*Pycnonotus goiavier*) within the focal area in a) Kuala Lumpur, b) Jakarta and c) Metro Manila.

Cells with high current density (black) indicate higher probabilities for Eurasian tree sparrow (*Passer montanus*) and Yellow-vented bulbul (*Pycnonotus goiavier*) movement between patch sites (Figs. 5 and 6 ). Cells with low current density (white) show portions of the landscape contributing the least to connectivity. The red lines represent the least-cost path while the yellow lines represent the input parameters of landscape resistance and focal node areas (vegetation density and patch size) (Figs. 5 and 6).

### 3.2 Connectivity analysis

There were 251 focal nodes calculated for connectivity analysis for Eurasian tree sparrow (*Passer montanus*) in Kuala Lumpur. The minimum distance is between site edge 81 and 82 (17 m) while the maximum distance is between 62 and 67 (997 m). In Jakarta, 160 focal nodes were calculated for connectivity analysis. The minimum distance is between site edge 1 and 2 (30 m) while the maximum distance is between 30 and 39 (999 m). In Metro Manila, 105 focal nodes were calculated. The minimum is between site edge 18 and 59 (30 m) while the maximum distance is between 241 and 247 (1000 m) (Table 5).

**Table 5:** Calculated straight-line distances between sources site edges for each species in Kuala Lumpur, Jakarta and Metro Manila.

Study area	Kuala Lumpur				Jakarta				Metro Manila			
Conefor input	Eurasian tree sparrow (*Passer montanus*)		Yellow-vented bulbul (*Pycnonotus goiavier*)		Eurasian tree sparrow (*Passer montanus*)		Yellow-vented bulbul (*Pycnonotus goiavier*)		Eurasian tree sparrow (*Passer montanus*)		Yellow-vented bulbul (*Pycnonotus goiavier*)	
	Min	Max	Min	Max	Min	Max	Min	Max	Min	Max	Min	Max
Patch site IDs	81, 82	62, 67	295, 71	35, 132	1, 2	30, 39	45, 46	230, 239	18, 59	15, 86	75, 76	71, 207
Straight-line distances (m)	17	997	28	999	30	999	13	1000	30	997	12	120

There were 295 focal nodes calculated for connectivity analysis for Yellow-vented bulbul (*Pycnonotus goiavier*) in Kuala Lumpur. The minimum distance is between site edge 295 and 71 (28 m) while the maximum distance is between 35 and 132 (999 m). In Jakarta, 611 focal nodes were calculated for connectivity analysis. The minimum distance is between site edge 45 and 46 (13 m) while the maximum distance is between 230 and 239 (1000 m). In Metro Manila, 340 focal nodes were calculated for connectivity analysis. The minimum is between site edge 75 and 76 (12 m) while the maximum distance is between 71 and 207 (120 m) (Table 5).

### 3.3 Least-cost models

**Fig. 7:**
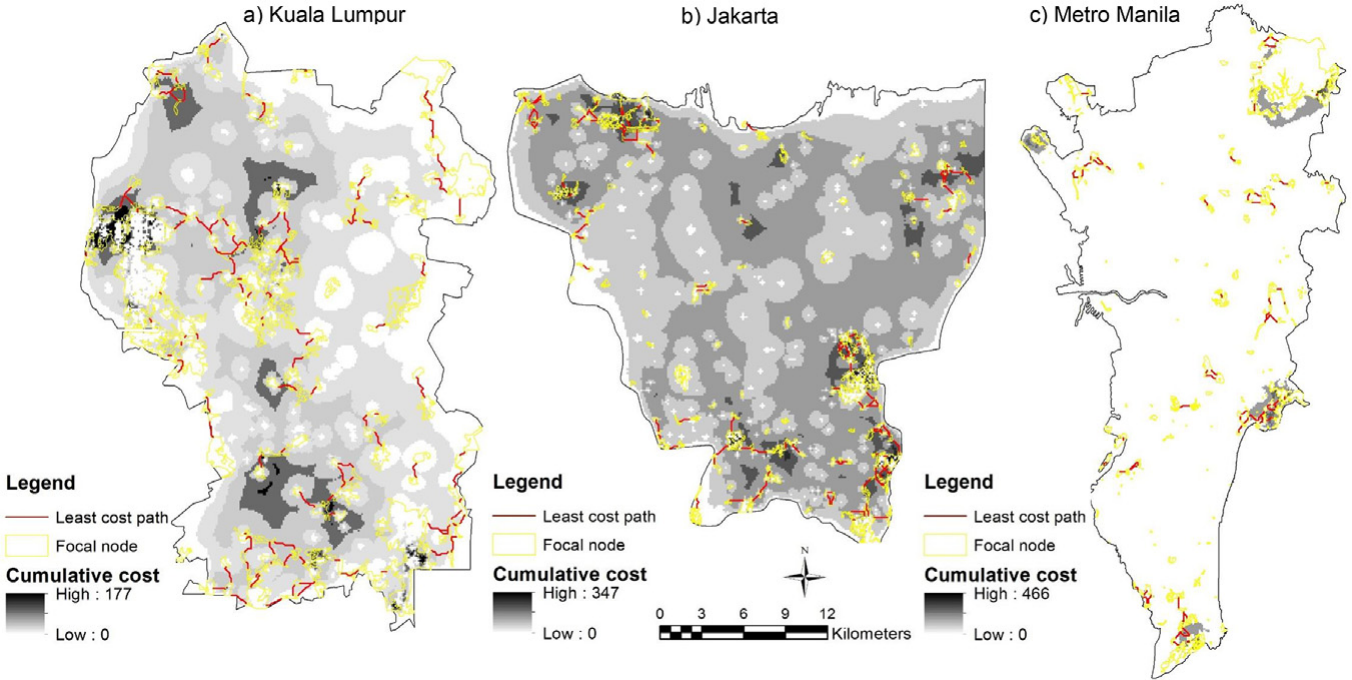
Cumulative cost and identified LCPs between patch sites for Eurasian tree sparrow (*Passer montanus*) within the focal area in a) Kuala Lumpur, b) Jakarta and c) Metro Manila.

**Fig. 8:**
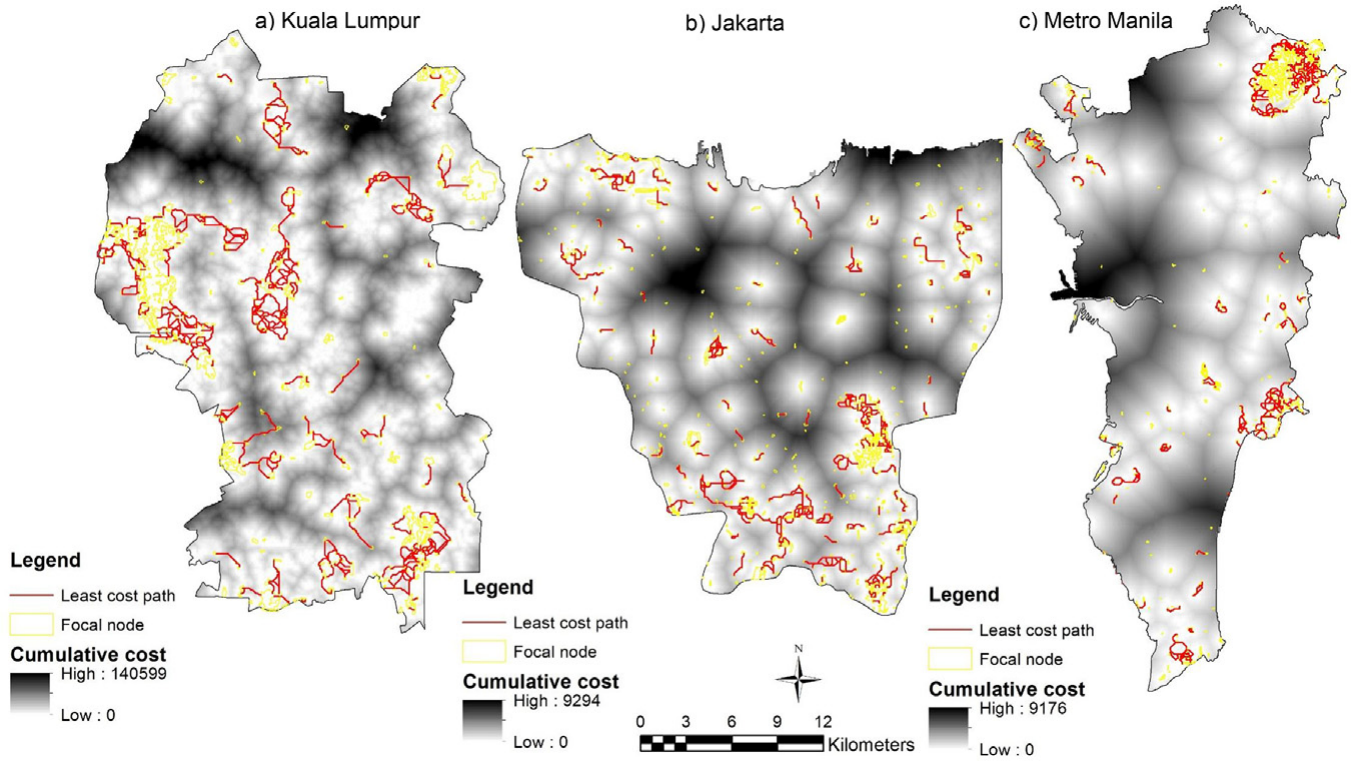
Cumulative cost and identified LCPs between patch sites for Yellow-vented bulbul (*Pycnonotus goiavier*) within the focal area in a) Kuala Lumpur, b) Jakarta and c) Metro Manila.

Least-cost models generate maps of the cumulative cost that highlight least-cost corridors and least-cost paths between patch sites. Cells with the lowest cumulative cost (white) define the least-cost paths (LCPs), represented in red line (Figs. 7 and 8 ). As for circuit model outputs, cells with low cumulative cost (white) show where species are more likely to move and cells with high cumulative cost (black) show portions of the least-cost corridors that contribute less to connectivity (Figs. 7 and 8).

### 3.4 Integrated models

Combined models show the current density within the corridors identified in the least-cost models and provide values of effective resistance, a connectivity measure complementing LCP lengths (Table 6). Only current density values within least-cost corridors are taken into account in combined models. In general, this means that smaller ranges of values have to be displayed, allowing critical connections on the map to be highlighted more accurately.

**Table 6:** Comparative Table of the straight-line distance (SLDis), least-cost path lengths (LCP length) and effective resistances (EffResist) resulting from the combined models for Eurasian tree sparrow (Passer montanus) and Yellow-vented bulbul (Pycnonotus goiavier) in three cities.

Study area	Link ID	Patch site ID 1	Patch site ID2	SLDis (m)	LCP length (m)	EffResist
Kuala Lumpur	Min	131	132	13	121	351
	Max	45	61	453	999	1053
Jakarta	Min	52	358	28	130	551
	Max	50	51	740	995	1833
Metro Manila	Min	101	121	40	106	402
	Max	60	61	860	998	1935

### 3.5 Ecological connectivity model 2030

**Fig. 9:**
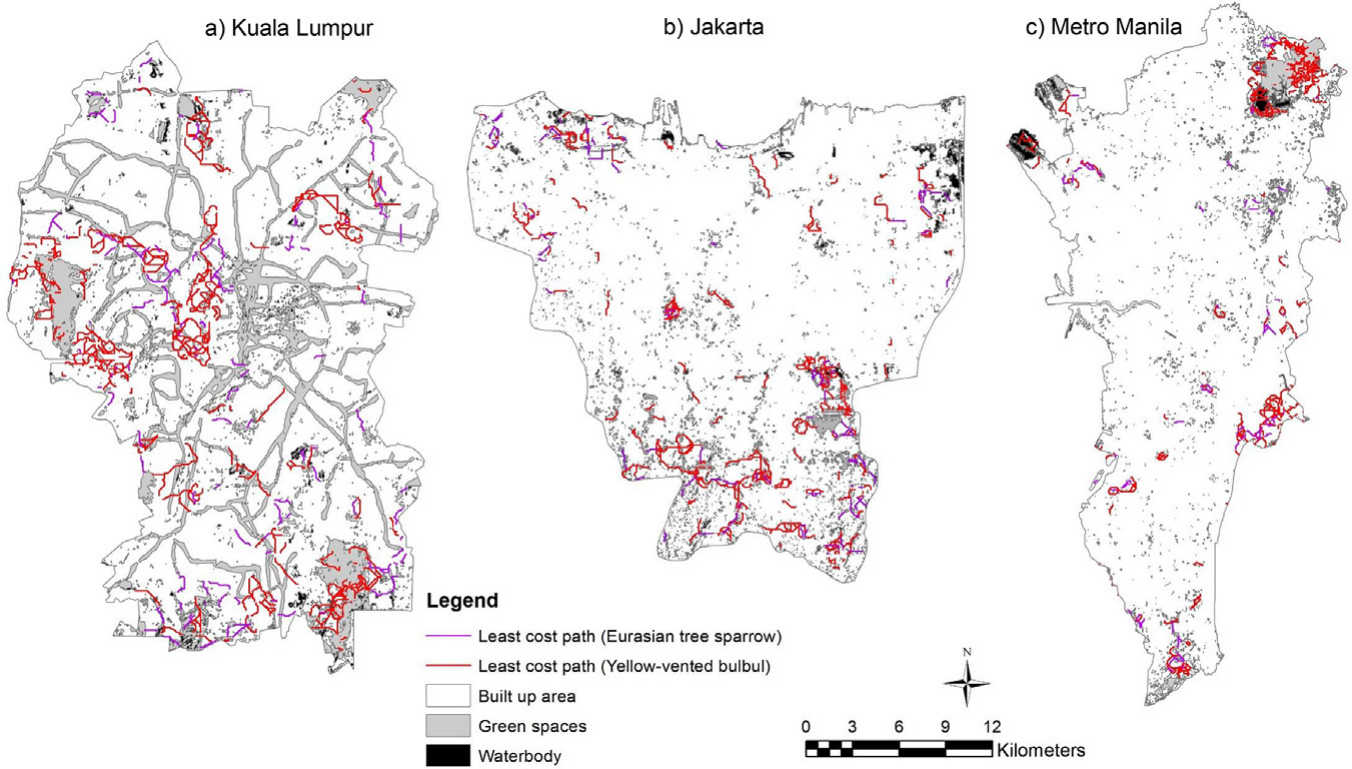
Ecological connectivity network 2030 in a) Kuala Lumpur, b) Jakarta and c) Metro Manila.

The ecological connectivity model for 2030 shows the combined least-cost paths of both species in three cities. This model can be used as guidance for future urban planning (Fig. 9).

## 4 Discussion

A novel integrated modeling approach combining circuit theory, connectivity and least-cost path analysis was used to identify the potential corridors to connect green space patches for ecological connectivity networks. The present study is among the first to present a novel integrated approach to identify and assess optimal corridors in urban environments under current and future development scenarios. In such a rapidly evolving, heterogeneous and highly fragmented landscapes, the identification of corridors which should be prioritised is important to better design, preserve and can improve ecological networks. These networks of multifunctional ecosystems are undoubtedly crucial for nature conservation and human well-being as well, since they support biodiversity, ecological processes and services in urbanised landscapes [1, 5].

This study used circuit theory which was parameterised with green space structures such as patch size and vegetation density to optimise corridor effectiveness for two bird species; Eurasian tree sparrow (*Passer montanus*) and Yellow-vented bulbul (*Pycnonotus goiavier*). Similar to the surrogate species approach adopted by earlier studies [14, 16], the present study employed two target species with different uses of landscape structure. This approach aims to optimise the continuity and conditions of green spaces within the study area so that opportunities for individual passage may be maximised for a wide range of species. The present study was based on the literature (Tables 3 and 4) which advocated that, species that are present within the identified habitat patches may benefit from the establishment of connective landscape features between them, if the composition of vegetation within such patches is sufficiently similar. As similar species may benefit to a greater extent from particular landscape attributes than others, the approach used here effectively aims to restore the condition of habitat and thus most likely to suit the individual requirements of the species present.

The model (Figs. 7 and 8) is significant in predicting bird density from ecological and structural connectivity through the use of foraging and nesting (vegetation density and patch size), and seed dispersal (patch distance) as indicative measuring variables. It extended the use of circuit theory and connectivity to build a spatially explicit model to understand habitat factors on biodiversity. The landscape structure factors can give an indication of the conditions of surrounding matrix and possible future change surrounding green spaces. Uezu et al. [16] demonstrate that species differ in their responses to fragmentation, and bird diversity and abundance are related to the structural and functional connectivity and patch size factors. For the Eurasian tree sparrow (*Passer montanus*), patch size was the main factor determining cumulative cost current density, while least-cost path was more affected by the degree of patch connectivity, the former by the presence of corridors and the latter by the distance between patches. On the contrary, vegetation density had no effect on the priority corridors of Eurasian tree sparrow (*Passer montanus*) and had a positive effect on Yellow-vented bulbul (*Pycnonotus goiavier*). This study emphasises the importance of considering species perceptions of landscape, especially functional connectivity, in developing priority corridors of ecological connectivity networks.

Circuit theory was selected because of its ability to provide rapid, repeatable results using the simple connectivity measure of resistance distance (distance metric) as the effective resistance between a pair of nodes [22]. A convenient property of the resistance distance is that it incorporates multiple pathways connecting nodes, with resistance distances measured between node pairs decreasing as more connections are added. The use of the model was also favoured as it evaluates sites on the basis of their ability to support a wide range of species, not only in areas containing significant habitat, but also in sites currently lacking vegetation. It must be noted that this methodology may not be as easily applied in less densely populated urban settings where differences in habitat condition are more subtle. However, many urban centres have already experienced comparable levels of modification, and as such, this methodology will be readily applicable to landscape planners in many regions [14].

In this study the model has proven that the circuit theoretic model was able to overcome the limitation of the least-cost model by simultaneously considering different suitable routes. This major advantage over the least-cost model has also been mentioned by other studies [20, 22]. The circuit model was also able to spot critical connections that contribute the most to network connectivity and to identify corridors with optimal connectivity (Figs. 5 and 6). These latter findings were similar to the one highlighted by the least-cost model but were more difficult to spot on maps, as observed in Rainey [61], which compared the least-cost and circuit analyses. In addition, this study highlighted an additional limitation of circuit theory approach. The approach is only effective in urban heterogeneous landscapes, as illustrated in the results for the Eurasian tree sparrow (*Passer montanus*) and Yellow-vented bulbul (*Pycnonotus goiavier*) in Metro Manila, for which a few optimal corridors were identified by the circuit model due to the presence of homogeneous areas (built up area) around the city (Figs. 5 and 6).

The least-cost model was the first and most popular method studied. Throughout the study, this has proven to be an effective way to calculate distances and to identify the most optimal routes between source sites (Table 5). This method also provides an easily understandable assessment of connectivity via the least-cost path length metric, which is a much easier way to interpret than accumulated-cost in terms of dispersal distance [62]. Nevertheless, the study has demonstrated that the least-cost model has also some constraints such as not considering all possible routes that could contribute to connectivity or providing connectivity assessments that are only related to a single, most cost-efficient route identified in a given landscape. These same limitations were pointed out previously by Mcrae and Beier [20].

The combined model benefits from the advantages of both least-cost and circuit models. In our study, the outputs generated via the combined model showed the outlines of the optimal corridors identified by the least-cost models and highlighted the critical connections within them with more precision (Table 6). It also provided an assessment of connectivity for each corridor via the least-cost path length metric. In addition, the combined model was able to compute the effective resistance for each least-cost corridor identified. This second connectivity metric complements the least-cost path length metric and reflects the contribution of alternative suitable corridors. The results suggest that planning for priority corridors should be developed at the link between patches which have low values of least-cost path lengths (LCP length) and effective resistances (EffResist), for example, Kuala Lumpur showed the lowest effective resistance value followed by Jakarta and Metro Manila (Table 6). Even though the combined model appears to be the ideal combination between least-cost and circuit models, it must be emphasised that the circuit models have to be processed in the first place in order to generate the combined model outputs, as well as to interpret them adequately.

The models generated for this study present a first approximation of connectivity for both species of ecological significance, an integrative approach towards structure and function of green space. The results indicate some of the challenges currently confronting both bird species, particularly at the source sites selected (Table 6). This study provides scientific implications and solutions to optimise the green space structure under rapid urban expansion and to ensure species’ persistence and connectivity of green space. As practical implications, ecological connectivity networks introduce a novel integrated methodological approach that can help planners and decision makers to design proper policy and urban planning for the cities and predict changes in avian biodiversity. Ecological connectivity networks can inform conservation planning for biodiversity and it can then indicate how urban planning can minimise ecological damage [17]. For social implications, green planning is known to have various psychological benefits. The ecological network is a decision support tool and thus incorporates public opinions, enhances social responsibility and enhances awareness of the broader benefits of green spaces.

This study provides recommendations to improve landscape connectivity for both species. This study was based on bird species but the method can be repeated on a range of animals from amphibians to mammals. It will improve the understanding of the use of integrated circuit theory and least-cost path models in connectivity assessment by using various target species with different dispersal distances and habitat requirement. It may promote the use of circuit theory among stakeholders from different backgrounds. The selection of appropriate landscape structure in this model will allow many applications, ease of calculation, functional basis, and simplicity of interpretation by a range of specialist and non-specialist stakeholders. Regardless, there continues to be a need for landscape metrics to calculate landscape structure because they are seen by many land managers and stakeholders as simple, intuitive tools for assessing and monitoring changes in landscape pattern and, by extension, the effects on underlying ecological processes. Future needs include: (1) the development of more user-friendly landscape analysis software that can simplify analyses and visualization; and (2) studies that clarify the strengths and weaknesses of different approaches, including the potential limitations and biases in modelling connectivity. In the future, they could be related to other datasets to provide a complete interpretation of ecological processes and phenomena. By replicating the methodological approach presented in this study, these results could also be used as initial data to predict how urban developments might affect the urban connectivity in rapidly expanding cities, either for birds or other animals.

## 5 Conclusions

This study sought to present a novel integrated approach to assess and model connectivity for two species in the studied cities in order to provide priority corridors for an ecological connectivity network. This study has: (1) developed predictive connectivity models for two focal species based on least-cost and circuit models; (2) identified priority corridors and assessed their connectivity and highlighted critical connections within them; and (3) provided recommendations to improve landscape connectivity for both species. The models used in this study have complementary approaches that can contribute to a more concrete assessment of the connectivity for biodiversity conservation and urban planning. This model also could be applied for human recreation using factors such as social, cultural and economic variables. Despite these limitations, our study has important implications for the design and management of landscape connectivity in Southeast Asian cities and possibly other similar tropical areas which experience rapid urban expansion. We can conclude that the popular least-cost model is an efficient and reliable method to identify corridors for which maintenance and improvement have to be prioritized to establish and implement ecological networks. The least-cost path lengths calculated by the least-cost models provide a convenient connectivity assessment that could explain the potential corridors for bird’s movement at one of the source sites. The circuit model, despite the fact that it has not been widely used yet in connectivity studies, has proved to be a valuable method complementing the least-cost model by highlighting alternative corridors and critical connections playing an important role in landscape connectivity. The circuit model has also shown its ability to highlight priority corridors similar to the ones identified by the least-cost model under rapid urban expansion. The combined model is an effective way of highlighting critical connections within the priority corridors identified by the least-cost model. It allows for the maintenance and improvement of existing corridors or for the creation of ecological networks in future planning. This study can help nature conservation and urban planning decisions to maintain or design appropriate ecological networks. The multistep framework of this study will allow other researchers to identify priority corridors in urban environments and quantify their connectivity.

## Declarations

### Author contribution statement

Amal N. M. Nor: Conceived and designed the experiments; Performed the experiments; Analyzed and interpreted the data; Contributed reagents, materials, analysis tools or data; Wrote the paper.

Ron Corstanje: Conceived and designed the experiments; Analyzed and interpreted the data; Wrote the paper.

Jim A. Harris: Wrote the paper.

Darren R. Grafius: Conceived and designed the experiments; Contributed reagents, materials, analysis tools or data; Wrote the paper.

Gavin M. Siriwardena: Conceived and designed the experiments; Wrote the paper.

### Funding statement

Amal N. M. Nor was supported by a scholarship of Academic Training Scheme (SLAI) from the Ministry of Education Malaysia and University Malaysia Kelantan. This work was supported by Fragments, Functions and Flows in Urban Ecosystem Services (F3UES) project as part of the larger Biodiversity and Ecosystem Service Sustainability (BESS) framework (grant number NE/J015067/1).

### Competing interest statement

The authors declare no conflict of interest.

### Additional information

No additional information is available for this paper.
